# Cancrum Oris

**Published:** 1896-10

**Authors:** J. P. Shaw


ARTICLE IV.

### CANCRUM ORIS.

BY J. P. SHAW, D. D. S.

Because of cancrum oris occurring so rarely in an adult I have prepared this paper. All writers treat this disease as one peculiar to children in crowded districts, where the lack of fresh air and healthful diet, together with scrofulous and syphilitic tendencies, act as predisposing causes, and cite only a few cases where gangrene occurs in adults.

It is not my intention, however, to attempt a treatise on cancrum oris, nor to embellish my paper with high-sounding phrases or technicalities, but as concisely as possible to detail a case as it came under my observation. This paper is a compilation of notes taken by myself and Dr. W. C. Keene, the physician in charge, and the case is as follows :

The patient was about thirty-five years old. For several years he had lived in Southern Missouri and in Arkansas, returning to this, his native State, a few months previous to his illness. His occupation was that of a "cropper," or farmer on shares, his life consequently an active one. His diet was very plain, consisting chiefly of corn-bread and pork. At this time he was apparently in good health.

About the last week in May he had a left inferior first molar extracted by a physician. A few days later he sent for the same physician, complaining of a sore mouth, for which no relief was afforded. On June 3d, Dr. W. C. Keen was called in, and later myself. An examination resulted in the diagnosis of gangrene, and the disease was found to be considerably advanced. The cheek, as well as the gum-tissue, was involved, the sloughing was very great, and the discharge offensive.

All of the part involved was thoroughly scraped and all appearance of diseased tissue removed, and the part was then well cauterized with nitrate of silver. A nourishing diet was ordered, and a tonic of iron and quinine prescribed. At the second visit the socket of the tooth, where the destruction had been greatest, was again scraped and an application of tinc. muriate iron made. A mouth-wash of tinc. myrrh, listerine, and Condy's fluid, was ordered to be used *ad libitum*. This course of treatment was followed by very happy results, as healthy granulation was established and a decided general improvement. June 10th the patient was dismissed, as he had sufficiently convalesced to resume his work on the farm.

On June 18th the case again required attention, and an examination showed that the disease had progressed to an alarming extent. An attempt was again made to remove the sloughing tissue, but it was fruitless, because of the extent, The former treatment was resumed as far as possible, but without any good results, the patient dying July 2d.

The general condition of the patient during the progress of the disease, will perhaps be interesting if not instructive. At no time was there a perforation of the cheek, and the extension of the disease was confined principally to the left side, although all the tissue of the oral cavity was affected. By introduction of the probe it was very apparent that the osseous tissue was not only denuded but must have been acted upon. It seemed as if the probe would extend almost to the orbit, but an examination, or any attempt to treat, the jaw was so painful that it had to be given up during the last few days, when the disease had progressed to its greatest extent. Because of the swelling and distortion nothing could be determined except by examination with an instrument. The gums extended over all the teeth in both the upper and lower jaws.

From the period of relapse the glands of the neck were swollen and the face greatly distorted. Later the glandular system throughout the entire body was involved, seeming to be greater on the left than on the right side.

The patient complained of fainting spells at night and of a constant severe headache. I cannot state the condition of the bowels, but heard no complaint of diarrhœa, as seems to be true of children similarly affected.

No hemorrhage occurred at any time except when diseased tissue was being scraped. There was a constant expectoration of a fibrous greenish discharge, which was unbearably offensive during the last few days.

Nothing could be ascertained from family history as a predisposing cause, and from all the indications this seems to have been a true case of cancrum oris.—*Dental Digest.*

